# Numerical Mechanistic Modelling of Drug Release from Solvent-Removal Zein-Based In Situ Gel

**DOI:** 10.3390/pharmaceutics15102401

**Published:** 2023-09-28

**Authors:** Setthapong Senarat, Pornsarp Pornsawad, Nutdanai Lertsuphotvanit, Jesper Østergaard, Thawatchai Phaechamud

**Affiliations:** 1 Programme of Pharmaceutical Engineering, Faculty of Pharmacy, Silpakorn University, Nakhon Pathom 73000, Thailand; senarat_s@silpakorn.edu; 2 Department of Mathematics, Faculty of Science, Silpakorn University, Nakhon Pathom 73000, Thailand; pornsawad_p@su.ac.th; 3 Program of Pharmaceutical Technology, Department of Pharmaceutical Technology, Faculty of Pharmacy, Silpakorn University, Nakhon Pathom 73000, Thailand; lertsuphotvanit_n@silpakorn.edu; 4 Department of Pharmacy, University of Copenhagen, Universitetsparken 2, DK-2100 Copenhagen, Denmark; jesper.ostergaard@sund.ku.dk; 5 Department of Industrial Pharmacy, Faculty of Pharmacy, Silpakorn University, Nakhon Pathom 73000, Thailand; 6 Natural Bioactive and Material for Health Promotion and Drug Delivery System Group (NBM), Faculty of Pharmacy, Silpakorn University, Nakhon Pathom 73000, Thailand

**Keywords:** zein, in situ forming gel, numerical simulation, mechanistic model, levofloxacin, drug release kinetics

## Abstract

The development of effective drug delivery systems remains a focus of extensive research to enhance therapeutic outcomes. Among these, in situ forming gels (ISG) have emerged as a promising avenue for controlled drug release. This research focuses on the mathematical modeling of levofloxacin HCl (Lv) release from zein-based ISG using the cup method, aiming to mimic the environment of a periodontal pocket. The drug release behavior of the ISGs was investigated through experimental observations and numerical simulations employing forward and central difference formula. Notably, the experimental data for drug release from the 20% *w*/*w* zein-based ISG formulations closely aligned with the simulations obtained from numerical mechanistic modeling. In summary, 20% *w*/*w* zein-based ISG formulations demonstrated nearly complete drug release with the maximum drug concentration at the edge of the matrix phase values consistently around 100–105%, while 25% *w*/*w* zein-based ISG formulations exhibited somewhat lower drug release extents, with values ranging from 70–90%. Additionally, the rate of drug transport from the polymer matrix to the external phase influenced initial release rates, resulting in a slower release. The utilization of glycerol formal as a solvent extended drug release further than dimethyl sulfoxide, thanks to denser matrices formed by high-loading polymers that acted as robust barriers to solvent removal and drug diffusion. Furthermore, UV-vis imaging was utilized to visualize the matrix formation process and solvent diffusion within the ISGs. The imaging results offered valuable insights into the matrix formation kinetics, controlled drug release mechanisms, and the influence of solvent properties on drug diffusion. The combination of mathematical modeling and experimental visualization provides a comprehensive understanding of drug release from zein-based ISGs and offers a foundation for tailored drug delivery strategies.

## 1. Introduction

Over the past few years, there has been significant attention on the development of drug delivery systems due to their potential to improve the therapeutic effectiveness and safety of pharmaceutical formulations [1,2]. Among various approaches, in situ forming gels (ISGs) have gained prominence as a promising strategy for controlled drug release [3]. Initially, an ISG consists of a solution containing polymers, organic solvents, and drugs, which solidifies into a drug-loaded polymeric matrix upon injection into an aqueous environment. This phase inversion process occurs due to thermodynamic instability, leading to the separation of the miscible organic solvent from the aqueous solution, thereby providing sustained and localized drug release over an extended period [4]. It is now possible to administer highly concentrated solutions of therapeutic drugs to the desired tissue with a reduced risk of adverse drug effects [5]. One of the critical areas where controlled drug release can have a significant impact is in the treatment of periodontitis, a chronic inflammatory condition affecting the tissues surrounding the teeth, including the gingiva, periodontal ligament, and alveolar bone [6]. Effective treatment of periodontitis often requires the localized delivery of therapeutic agents, such as antimicrobial drugs, into the periodontal pocket—a unique microenvironment within the oral cavity [7]. The microenvironment of the periodontal pocket presents several challenges, including limited space, constant exposure to saliva, and the need for sustained drug release [8]. ISGs are designed to be injected directly into the periodontal pocket, where they quickly solidify and release therapeutic agents in a controlled manner [9,10]. Nevertheless, the clinical application of ISGs is currently hindered by the lack of predictable drug release dynamics, which heavily rely on the matrix formation processes [11,12].

The drug release kinetics from controlled release implants are impacted by numerous variables such as polymer swelling, polymer erosion or degradation, implant shape or surface area, drug dissolution, and water inflow rate [13]. To accurately design drug-delivery devices, a comprehensive mathematical model should incorporate all relevant parameters. Diverse release models are examined to comprehend drug release processes and predict the quantity of drug released over time. In controlled systems, the burst drug release effect, characterized by an initial rapid release, is frequently observed, followed by sustained release. Release kinetics are utilized to analyze and match the release profile, providing a representation of the release behavior in controlled release systems [14,15].

Empirical models, although simple, do not account for the dynamic responses of actual drug delivery devices, making them inadequate for accurately simulating the impact of device design variables on drug release. Mechanistic models, on the other hand, incorporate mass transport and chemical phenomena such as polymer swelling, water influx, drug dissolution, matrix void space creation, matrix degradation, and convective processes. These comprehensive models enable precise forecasts of drug release kinetics from specific drug delivery systems. Complex drug delivery systems can be depicted by models that incorporate non-linear differential equations with multiple variables and parameters, which require numerical solutions to accurately capture their kinetics [15].

Wang et al. constructed a mechanistic model to study the controlled release of β-lapachone (β-lap), a poorly water-soluble anti-cancer agent, from cylindrical pre-formed polymer millirod implants [16]. The model considered various chemical and mass transport processes, including water influx, pore creation, excipient-drug complex formation, crystalline drug dissolution, and diffusion in solid and liquid phases. The accuracy of the model was validated by comparing its outputs to experimental data and obtaining optimal parameter estimates. This validated model enabled the prediction of how the enhanced solubility of β-lap affects drug release kinetics, allowing simulation of the impact of various excipients or drug loading dosages on drug release [15]. Moreover, Raman et al. employed a mechanistic model to examine the release kinetics of lysozyme from a fast phase inverting in situ gel [17]. The model employed one-dimensional diffusion reaction equations to represent drug dissolution, water input, solvent efflux, and drug diffusion. The implant consisted of a polymer phase with dispersed drug and a water phase. The simulations demonstrated various drug release kinetics, including zero-order and initial burst release, through the manipulation of model parameters [17].

The previous mechanistic and mathematical models of ISG have a limitation as rapid phase inversion of the implant (within seconds to minutes) is assumed, whereas in reality most ISGs form spherical or globular shapes that undergo phase inversion over longer time scales (spanning from hours to days) in both in vivo and in vitro environments [16,18,19]. The rate of implant phase inversion significantly impacts the drug release kinetics [20]. Therefore, alternative mechanistic models are required to accurately depict the drug release kinetics from realistic globular implants that undergo slower phase inversion. These mechanistic models can be utilized to simulate drug release from thin in situ forming membranes in controlled in vitro settings [19,21,22]. In addition, the utilization of mathematical modeling with MATLAB^®^ software was implemented to study drug delivery from single-layer matrix-type vaginal rings with a torus-shaped structure, employing the pseudo-steady state approximation (PSSA) [23]. The reliability and utility of this model was validated through the comparison of simulation results with experimental release data documented in the literature

Numerical techniques are commonly employed to approximate and analyze the drug release characteristics of in situ gels [24]. By employing the numerical technique to discretize the differential equations that govern drug diffusion and gel degradation, researchers can simulate and evaluate the spatiotemporal drug release profiles from these gels [25]. This approach may facilitate a comprehensive understanding of the drug release kinetics, optimization of gel formulations, and prediction of therapeutic efficacy, ultimately contributing to the development of efficient and tailored ISG-based drug delivery strategies [24,25].

Among in situ forming matrices, there has been considerable interest in zein-based solvent removal phase-inversion systems [9]. Zein, a plant-derived protein extracted from maize, has demonstrated immense potential as a versatile biomaterial for various biomedical and pharmaceutical applications [26,27]. Zein has received a Generally Recognized As Safe (GRAS) approval from the US-FDA, making it suitable for various industrial and consumer applications, including plastics, molded products, inks, chewing gum, printing film, food coatings, and paper [28]. In the biomedical field, zein is extensively studied for its potential in the controlled drug delivery of small molecules, production of bioactive membranes, and the creation of 3D scaffolds for tissue regeneration through the use of micro- or nano-structured templates [29]. In the context of this research, zein holds great potential as a matrix forming component for the development of a solvent removal phase inversion-based ISG system loaded with antimicrobial agents for the treatment of periodontitis [9,30]. Moreover, the application of a pharmacokinetics mathematical model based on a partial differential equation for doxorubicin-loaded zein in situ gel in tumors facilitated the understanding of the release and action mechanisms, specifically through the dynamic balance between diffusion and elimination, allowing for predictions of local doxorubicin concentrations in tumor tissue [31]. Mathematical modeling was employed to evaluate the mechanism of 5-azacytidine release from zein protein nanoparticles. Real-time release kinetics were supported by DDsolver 1.0 software, which analyzed the release kinetics using various models, including Zero order, First order, Makoid–Banakar, Higuchi’s, Weibull, Hixson–Crowell, Korsmeyer–Peppas, Hopfenberg, Baker–Lonsdale, and Gompertz kinetics [32]. The Korsmeyer–Peppas model showing the best fit (highest r^2^ value) indicated that drug release was controlled by a diffusion mechanism and polymer relaxation [33].

**Figure 1:**
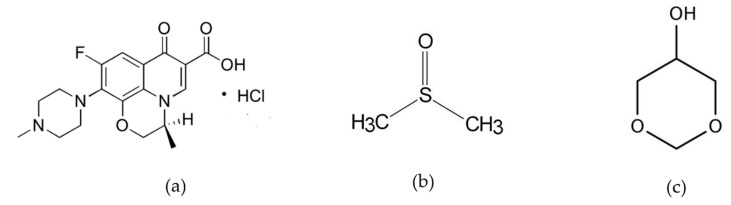
The molecular composition of levofloxacin HCL (Lv) in panel (**a**), dimethyl sulfoxide (DMSO) in panel (**b**), and glycerol formal (GF) in panel (**c**).

Levofloxacin HCl (Lv) is an antibiotic belonging to the third-generation fluoroquinolone class, known for its broad-spectrum activity by targeting bacterial DNA gyrase and topoisomerase IV, essential enzymes involved in DNA replication [34]. Lv has a molecular weight of 397.8 (Figure 1a) and it is freely soluble in water [35]. Lv demonstrates effectiveness against a wide range of aerobic Gram-positive and Gram-negative bacteria, and it also shows moderate activity against anaerobes [36]. In relation to modeling/simulation of drug transport, a model utilizing Fick’s second law of diffusion was developed to quantify the antibiotic concentration in levofloxacin-impregnated catheters, ensuring the maintenance of minimum inhibitory concentration (MIC) throughout catheter use, and it was predicted that similar results could be achieved with other antibiotics in the same class of molecules [37].

UV-vis imaging is an analytical technique that has been applied to the characterization of drug release behavior of ISG. UV-vis imaging allows researchers to visualize and monitor the behavior of these gels in real-time by capturing the absorption of ultraviolet-visible (UV-vis) light. By employing UV-vis imaging, various aspects of ISG can be investigated, including gel formation and drug distribution [38,39,40]. This non-invasive and quantitative imaging technique may provide valuable information, facilitating the understanding of the performance and behavior of ISGs, thus aiding in the development and optimization of drug delivery strategies.

This paper emphasizes the significance of comprehending the underlying mechanism of drug release from zein-based ISGs dissolved in dimethyl sulfoxide (DMSO) (Figure 1b) and glycerol formal (GF) (Figure 1c), while also addressing the challenges in modeling their release behavior. A numerical simulation based on mathematical model is performed to predict the drug release pattern, which can be correlated with the characteristics of matrix forming using UV-vis imaging technique. Additionally, the use of zein as the primary component for creating a matrix-based formulation has not been documented before in the context of developing a numerical technique for establishing a mechanistic model of drug release.

## 2. Materials and Methods

### 2.1. Materials

Zein (lot no. 9010-66-6) was purchased from Qingdao Sigma Chemical Co., Qingdao, China. The provision of Lv, which served as the antimicrobial drug, was graciously supplied by Siam Pharmaceutical Co., Bangkok, Thailand. DMSO (≥99.9%, Lot No. 1862992, Fisher Chemical, Horsham and Loughborough, UK) and GF (≥98.0%, Lot # BCCD7726, Sigma-Aldrich, Overijse, Belgium) were utilized as the solvents in the experimental procedures. Potassium dihydrogen orthophosphate (lot no. E23W60) and sodium hydroxide (lot no. AF310204) were purchased from Ajax Finechem, New South Wales, Australia, and used for the preparation of the phosphate-buffered saline (PBS).

### 2.2. Preparation of In Situ Forming Gel

ISGs containing zein at varying levels from 20% to 25% (*w*/*w*) were formulated by dissolving zein in DMSO and GF. The solutions were prepared by continuous mixing employing a magnetic stirrer. Furthermore, 1% (*w*/*w*) Lv was incorporated into the zein solutions. Table 1 presents a summary of the components included in each formulation.

### 2.3. In Vitro Drug Release Studies

The release characteristics of the drug from the created ISGs consisting of 1% (*w*/*w*) Lv from zein-based ISGs dissolved in DMSO and GF were examined. In order to mimic the drug release into the periodontal pocket, a cylindrical porcelain cup with a 1 cm diameter and a 1.2 cm height was used. The porcelain cup was filled with 0.4 g of the formulation and immersed into 80 mL of pH 6.8 PBS at 37 °C. A shaking incubator (NB-205, N-Biotek, Bucheon, Korea) was set to a speed of 50 rpm. To assess the drug release, 5 mL samples of the release medium were collected at specific time segments. After each sampling, an equal volume of fresh PBS was added to maintain a constant volume. The quantity of Lv released was measured at a wavelength of 287 nm using a UV-visible spectrophotometer (Cary 60 UV-Vis, Model G6860A, Agilent, Selangor, Malaysia). The detection of solvent absorbance was measured at 214 nm (A), meanwhile the visible imaging at 525 nm was applied for tracking zein matrix formation. The cumulative drug release was calculated as a percentage. All experiments were conducted in triplicate.

### 2.4. Model Development

#### 2.4.1. Characteristics of Depot Formation

**Figure 2:**
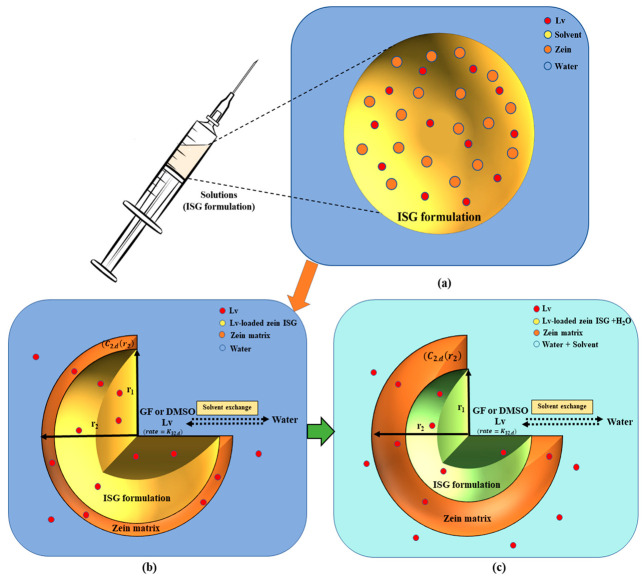
The schematic representation provides an overview of the mechanistic model and the sequential steps involved in the creation of solvent-removal zein-based ISG: Prior to injection, an ISG is composed of a solution comprising polymer, organic solvent, and drug (**a**). Upon ISG formulation injection, the phase-inversion process is initiated by solvent exchange, the polymer intentionally designed to be insoluble in water (**b**), undergoes solidification and transforms into a solid-like matrix (**c**). A schematic of the Lv-loaded zein ISG release model illustrates an axially symmetric model geometry with three different phases: the inner non-phase inverted state (r < r_1_), the polymer matrix phase (r_1_ < r < r_2_), and the bath solution phase. The diagram includes *C*_2,*k*_, which represents the drug concentration at the edge of the matrix phase, and *K*_32,*k*_, which represents the drug permeability coefficient between the polymer matrix and the exterior phase.

Based on previous studies, a mathematical model was employed to depict to illustrate the mass concentration changes of solvent, polymer, drug, and water through the matrix precipitation and phase inversion process [41,42]. The release model for Lv-loaded zein ISG assumes a spherical system with three phases: the interior liquid phase (1), the solid polymer matrix phase (2), and the exterior bath solution phase (3), as shown in Figure 2.

Following the injection of ISG formulations containing solvent, polymer, and drug into an aqueous solution, the phase-inversion for ISGs is initiated. This process leads to the immediate formation of a thin shell composed of a polymer matrix with continuous void space. This void space refers to the open regions within the matrix structure where the solvent was initially present. The shell envelops an interior core of the implant solution, which remains in a non-phase inverted state, resulting in an initial burst of drug release [41,42]. This phenomenon is depicted in Figure 2. The solvent and drug move outward from the interior to the exterior by passing through the porous matrix, while water diffuses from the exterior to the interior within the void fraction. Once the water surpasses a certain threshold, the polymer precipitates at the interface between the array and the solution, leading to the enhanced formation of the polymer matrix. As time progresses, the volume of the matrix phase gradually increases, causing the interior phase to swell due to water diffusion. Ultimately, the polymer completely precipitates, generating a matrix that surrounds an interior region filled with water [39,43,44,45].

#### 2.4.2. Assumptions in Modeling Drug Release

In developing our mathematical model for drug release from zein-based ISG, certain assumptions were made to simplify the complex process. These assumptions are crucial for understanding the context in which the model operates and interpreting the results accurately. Our model is based on the following assumptions:

Homogeneous distribution: It was assumed that Lv, zein, and the selected solvents (DMSO or GF) were homogeneously distributed within the gel matrix [46].

Neglect of convective fluxes: Convective fluxes within the gel matrix were neglected [47]. This assumption was made to focus on the diffusion-dominated drug release process and reduce model complexity.

Constant drug diffusion coefficient: The diffusion coefficient of Lv was assumed to be constant throughout the release process [48].

Spherical release pattern: In our study, we assumed a spherical release pattern for practical reasons, despite the gel’s cylindrical appearance within the cup [49].

First-order erosion: Matrix erosion was assumed to follow a first-order kinetic equation. This simplification was based on observed trends but may not capture all nuances of erosion behavior [50].

These assumptions provided a foundation for our model, enabling us to investigate the drug release process within the scope of this study. While they simplify the mathematical representation, it is important to recognize their limitations and the potential impact on the model’s accuracy. Further research may explore more detailed and complex models to refine our understanding of drug release from zein-based ISGs.

#### 2.4.3. Model of Transport Processes and Numerical Implementation

The diffusion parameters for levofloxacin HCl were determined within the framework of a one-dimensional axially symmetric simulation, in alignment with the methodology elucidated in the academic manuscript authored by Ravi Bhasker Patel [42].

##### Interior Phase

The concentration of the drug within the internal depot phase undergoes change as a function of both radial distance and time, governed by the following diffusion equation

(1) $$\frac{\partial C_{1,k}}{\partial t}=\frac{D_{1,k}}{r^{2}}\frac{\partial }{\partial r} [r^{2}\frac{\partial C_{1,k}}{\partial r}]\;$$

where $C_{1,k}$ represents the drug concentration at the interior phase, r is the radial distance, t is time and $D_{1,k}$ denotes the diffusivity of drug k through the interior phase.

Spherical symmetry allows for the description of conditions at the center as follows:

(2) $$r=0\;:\;\frac{\partial C_{1,k}}{\partial t}=0$$

The drug concentrations and transport rates at the interface between the interior liquid phase and void space of solid polymer matrix phase are continuous. Thus

(3) $$r=r_{1}\left(t\right):\;C_{1,k}=C_{2,k}\;;\;D_{1,k}\frac{\partial C_{1,k}}{\partial r}=\varepsilon D_{2,k}\frac{\partial C_{2,k}}{\partial r}\;$$

where $C_{2,k}$ signifies the drug concentration within the matrix phase, $D_{2,k}$ represents the diffusion coefficient of species k, and ε corresponds to the void fraction, denoting the ratio of free space to the total space within the matrix.

##### Matrix Phase

The drug is enclosed within the void space of the matrix phase. As a result of diffusion occurring in this void space, species concentrations exhibit radial and time variations as follows:

(4) $$\frac{\partial C_{2,k}}{\partial t}=\frac{D_{2,k}}{r^{2}}\frac{\partial }{\partial r} [r^{2}\frac{\partial C_{2,k}}{\partial r}]\;$$

where $D_{2,k}$ represents the drug’s diffusion coefficient. At the initial stage, within the void space of the thin matrix shell, the changes in drug concentrations are assumed to demonstrate a linear relationship with respect to radial position [42].

(5) $$t=t_{0}\;:\;C_{2,k}\left(r,t_{0}\right)= [C_{3,k}\left(t_{0}\right)-C_{1,k}\left(t_{0}\right)] [\frac{r-r_{1}\left(t_{0}\right)}{r_{2}\left(t_{0}\right)-r_{1}\left(t_{0}\right)}]+C_{1,k}\left(t_{0}\right)$$

At the outer surface, $r_{2}\left(t\right)$, the rate of species transport between the polymer matrix and the exterior phase is equivalent to the rate of species diffusion within the polymer matrix.

##### Exterior Phase

In the exterior phase, the water concentration (*C*_3,w_) and phase volume (*V*_3_) remain constant due to the excess of water present in the external environment. The rate of mass change for both the solvent and drug species in the external phase is equal to the rate of species transport that takes place between the polymer matrix and the external phase. Thus

(6) $$\frac{d}{dt}C_{3,k}+\frac{K_{32,k}}{V_{3}}C_{3,k}=\frac{K_{32,k}}{V_{3}}C_{2,k}\left(r_{2}\right)$$

where $C_{3,k}$ signifies the drug concentration at the exterior phase, $(C_{2,k}\left(r_{2}\right)$ represents the maximum drug concentration at the edge of the matrix phase, and $K_{32,k}$ denotes the drug permeability coefficient between the polymer matrix and the exterior phase.

Using the initial condition, $C_{3,k}\left(t_{0}\right)=0,$ the particular solution of Equation (6) is given by:

(7) $$C_{3,k}\left(t\right)=C_{2,k}\left(r_{2}\right)-C_{2,k}\left(r_{2}\right)e^{\frac{K_{32,k}}{V_{3}}t_{0}}\times {\;e}^{\frac{-K_{32,k}}{V_{3}}t}=C_{2,k}\left(r_{2}\right)[1-e^{\frac{K_{32,k}}{V_{3}}(t_{0}-t)}].\;$$

The drug concentrations exhibit a progressive increase and approach the value of $C_{2,k}\left(r_{2}\right)$ at a rate of $\frac{K_{32,k}}{V_{3}}$. This section aims to determine the rate $\frac{K_{32,k}}{V_{3}}$ and the value $C_{2,k}\left(r_{2}\right)$ when the drug concentration $C_{3,k}\left(t\right)$ at a given time is provided. However, the nonlinearity of the function on the right-hand side of Equation (7) poses challenges for inversion. In this case, the numerical technique can be applied to the ordinary differential equation (ODE) [51]. For convenience, the following notations are introduced:

$$y\left(t\right)=C_{3,k}\left(t\right),a=\frac{K_{32,k}}{V_{3}}\;\mathrm{and}\;b=\frac{K_{32,k}}{V_{3}}\;C_{2,k}\left(r_{2}\right)$$

Equation (6) is rewritten as:

(8) $$\frac{dy}{dt}+ay=b$$.

The derivative can now be discretized using a finite differences method. This can be accomplished by referring to the relevant section in the Supplement Data S1 that discusses the forward difference formula of order *O*(*h*) and the central difference formula of order *O*(*h*^4^) [52]. The following subsection presents the discretization strategy employed to calculate the parameters a and b.

Using the derived solution, the parameters $C_{2k,}\left(r_{2}\right)\;\mathrm{and}\;\frac{K_{32,k}}{V_{3}}$ can be calculated when the drug concentration $C_{3,k}\left(t\right)$ at a given time is provided.

### 2.5. Numerical Simulation

#### 2.5.1. Simulation of In Vitro Drug Release Profile

To examine the correlation between the drug concentration $C_{2,k}\left(r_{2}\right)\;$ at the edge of the matrix phase and the transport rate $\frac{K_{32,k}}{V_{3}}$ of the drug between the polymer matrix and the exterior phase, in relation to drug release patterns, the drug release data, represented as the concentration of drug in the aqueous medium at time ($C_{3,k}\left(t\right))$, is used to substitute in Equations (S3) and (S8). Then, the parameters as $C_{2,k}\;$ and $K_{32,k}$ are calculated by using numerical method as discussed above. The numerical method is implemented through an Octave software (GNU Octave software version 8.2.0 (2023)) [53]. The scripts for the forward difference formula employed in this study are given in Supplement Data S2, in addition to the scripts for the central difference formula to approximate the derivative.

#### 2.5.2. Investigation of Drug Release Profile Variations

This subsection focuses on simulating drug release profile by manipulating the rate of drug transport from the polymer matrix to exterior phase ($K_{32,k}$) and the maximum drug concentration at the edge of the matrix phase ($C_{2,k}\left(r_{2}\right)$). A comprehensive list of model parameters and their corresponding values for simulation is provided in Table 2. The parameter values were obtained by solving the ordinary differential equation of the model simulations, using the parameter range specified in Table 2. Additional parameters associated with drug release from the ISG were estimated based on this mechanistic model. These parameters include the rate of drug transport from the polymer matrix to the exterior phase ($K_{32,k}$), and the maximum drug concentration at the edge of the matrix phase ($C_{2,k}\left(r_{2}\right)$), as described in Equation (7). By utilizing the rate of drug transport and the drug concentration at the edge of the matrix phase listed in Table 2, the scripts provided in Supplement Data S2 enable the generation of the drug release profile.

### 2.6. UV-vis Imaging

**Figure 3:**
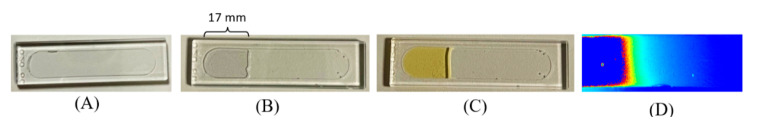
Schematic representation of the demountable quartz cell setup for UV-vis imaging: (**A**) Demountable cuvette quartz cell filled with 0.6% agarose gel, (**B**) Gel removed and replaced with PBS and sample, (**C**) Sample in contact with agarose gel inside the cuvette, (**D**) Absorbance map of recorded image analyzed using SDI Data Analysis software version 2.0.

A hot agarose solution (0.6%, *w*/*v*) was prepared by dissolving 0.6 percent agarose in boiling PBS (pH 6.8), the solution was pipetted into a demountable quartz cell (8.0 × 40.0 × 0.2 mm^3^ (W × L × H)) (Starna Scientific Ltd., Hainault, Essex, UK), which was subsequently closed. After 2 h at room temperature allowing for gelation, a part of the gel (17 mm from the edge of the cell) was cut away providing a straight interface (Figure 3). PBS was pipetted into the void occurring upon removal of gel and the cell was closed. The quartz cell with PBS as reference solution was placed in the D200 ActiPix UV-vis imaging system (Paraytec Ltd., York, UK). Imaging was performed at 214 nm, 280 nm, and 525 nm. After the collection of reference images with PBS, the sample (i.e., the in situ forming solution) was placed in the cavity (void) previously occupied by the PBS and imaging of the drug release processes commenced. In addition to the ISG formulations in Table 1, 0.1% (*w*/*w*) Lv was incorporated into the zein solutions comprising DMSO and GF to assess the effect of drug concentration. The images were recorded with 3 frames per min (file subsample 50) for 12 h in a temperature controlled (at 25 °C) cabinet shielding from outside light. The images were recorded using y Actipix D200 Acquisition program (Paraytec Ltd., York, UK). The images captured were analyzed and interpreted using the SDI Data Analysis software (version 2.0., Paraytec Ltd., York, UK).

## 3. Results and Discussion

### 3.1. Simulation of In Vitro Drug Release Profile

**Figure 4:**
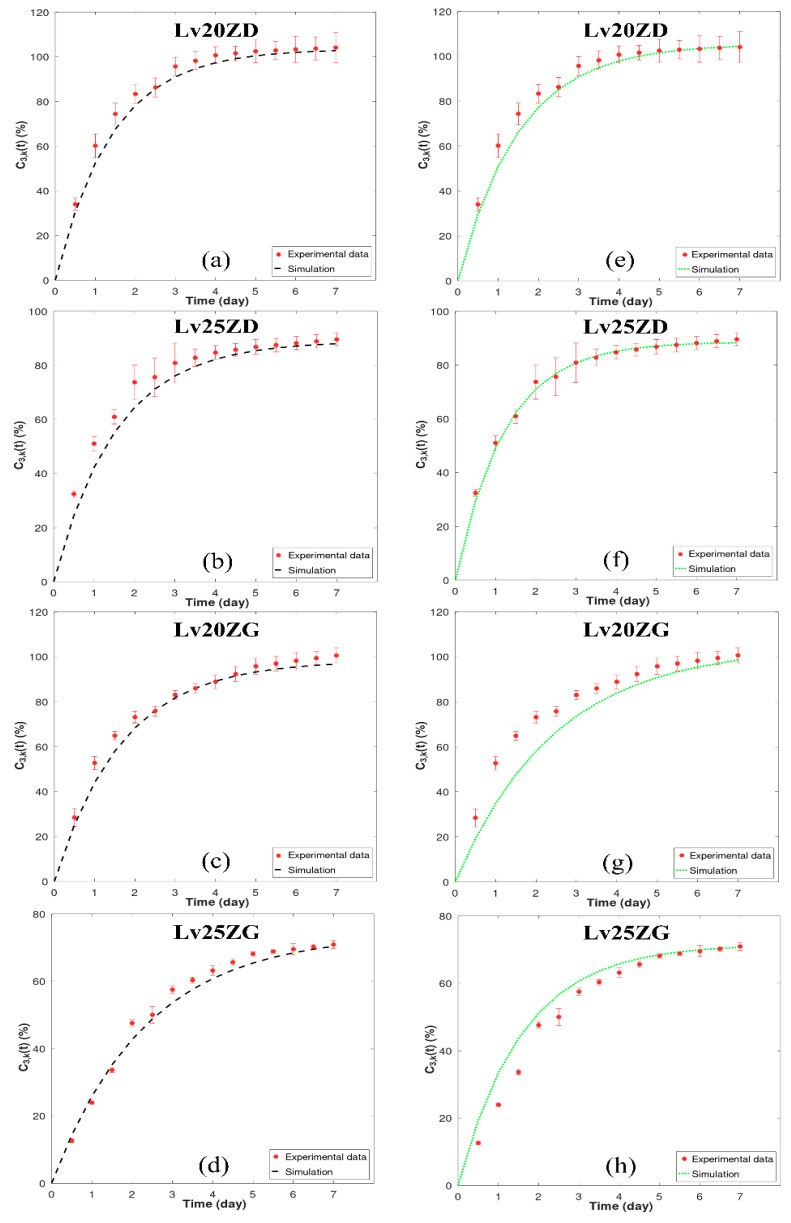
Model fit to experimental Lv-release data of zein-loaded ISG formulations by numerical differentiation: forward difference formula of order *O*(*h*) (**a**–**d**) and central difference formula of order *O*(*h*^4^) (**e**–**h**). The *y*-axis ($C_{3,k})$ represents the % cumulative of Lv released over time. The corresponding relative errors for (**a**–**d**) for *O*(*h*) were found to be between 1.34 and 1.52 The corresponding relative errors for (**e**–**h**) for *O*(*h^4^*) were found to be between 1.29 and 1.55, respectively.

The primary focus of this study revolves around the mathematical modeling of drug release from drug-loaded zein ISGs using the cup method, aiming to replicate the environment of a periodontal pocket [54]. Earlier investigators utilized this approach to examine the drug release characteristics of in situ forming drug delivery systems [55,56,57]. To comprehensively assess the drug release characteristics of in situ forming systems, the critical influence of zein concentration and solvent selection was considered. “Figure 4 presents observed drug release profiles from four distinct Lv-loaded formulations: 20% zein in DMSO (LvZD20), 25% zein in DMSO (LvZD25), 20% zein in GF (LvZG20), and 25% zein in GF (LvZG25). The observed drug release from the ISG is presented in Figure 4, displaying the percentage of drug amount released (% relative to 40 mg) over time (days). The drug release data from experimental observations over 12 h to 7 days were compared with the simulation results obtained using numerical differentiation methods: forward difference formula of order O(h) and central difference formula of order *O*(*h*^4^). These comparisons are shown in Figure 4a–d and Figure 4e–h, respectively.

Upon analysis, it was observed that the simulation results for LvZD20 and LvZD25 are closely aligned with the experimental data, as evident in Figure 4c,d,g,h. Conversely, the simulation outcomes employing order O(h) for LvZG20 and LvZG25 demonstrated better agreement with the experimental data when compared to the simulations utilizing order *O*(*h*^4^). In a parallel manner, researchers developed a mechanistic model to evaluate and predict drug release kinetics from millirods [16]. Their model provides invaluable insights into the intricate nature of drug-loaded millirod systems, unveiling the mechanisms that enable a rigorous quantification of the processes governing drug release from a polymer matrix.

In Table 2, it was observed that LvZD20 and LvZG20 exhibited nearly 100% Lv content, with $C_{2,k}\left(r_{2}\right)\;$ (maximum drug concentration at the edge of the matrix phase) ranging from 98.35% to 105.50%. Conversely, LvZD25 and LvZG25 displayed Lv content around 70% and 90%, respectively, with $C_{2,k}\left(r_{2}\right)$ values ranging from 71.58% to 88.97%. As the concentration of zein increased, there was a decrease in the rate of LV release, which was further prolonged by the inclusion of GF. The denser matrices formed by high-loading polymers effectively extended the duration of ISG drug release by establishing a robust barrier against solvent removal and drug diffusion [58,59]. Additionally, the values of $K_{32,k}$ (rate of drug transport from the polymer matrix to exterior phase) for LvZD20 and LVZD25 ranged from 32.55 to 50.23 (day^−1^), while in LvZG20 and LvZG25, they ranged from 51.57 to 64.76 (day^−1^). This implies that LvZD demonstrated a higher rate of drug transport from the zein matrix to the exterior phase compared to LvZG [29] The ISG composed of the less viscous DMSO series exhibited enhanced water penetration, facilitating accelerated drug release into the medium compared to the GF series ISG [9]. The viscous zein gel formation over time effectively delayed initial drug diffusion before transforming into zein matrices [60].

Furthermore, the parameter values from Table 2 were utilized to estimate the drug release equation, as presented in Equation (7), and the results are shown in Table 3. The simulation of drug transport using this model allowed for a reasonable approximation of drug release from Lv-loaded zein ISGs filled in the periodontal pocket.

In addition to providing Lv transport parameters, this work represents a methodological improvement with potential future applications. The numerical differentiation methods have presented a versatile approach. Specifically, the forward difference formula of order *O*(*h*) and the central difference formula of order *O*(*h*^4^) offer distinct advantages over analytical solutions. These methods are particularly beneficial when dealing with arbitrary geometries or functions that pose challenges for analytical expression. These developments could potentially offer advantages in upcoming investigations and efforts to enhance drug delivery techniques.

Model validation is indeed a crucial aspect of any modeling study. In our manuscript, we have incorporated model validation through the calculation of relative error (err) in Table 3. Relative error is a common metric used to assess the accuracy of our numerical simulations by comparing them to experimental data. It provides a quantitative measure of dissimilarity between the simulated data (y_app) and the measured data (y), and it is expressed as $\;\mathrm{err}\;=\;\mathrm{norm}\left(y\;-\;y\_\mathrm{app}\right)\;/\;\mathrm{norm}\left(y\right)$. In Table 3, we present the relative error values for our simulations. It is noteworthy that the relative error in the model approximates 1.5%. This value is generally considered quite low, indicating a high level of agreement between the model and experimental observations. A relative error value close to zero suggests a strong alignment between the two datasets, further affirming the accuracy of our modeling approach. Moreover, in our prior article [9], the analysis distinctly revealed the dominance of the first-order kinetic model, which exhibited a superior fit to the experimental dataset. Importantly, the consistently high goodness of fit (r^2^) values, approaching unity, provided substantial evidence in support of this observation for all Lv-loaded zein-based ISGs tested with varying solvents. Furthermore, our zein-based model, as delineated in Table 3 with r^2^ (*O*(*h*)) and r^2^ (O(*h^4^*)), closely paralleled the experimental data, reaffirming its accuracy in characterizing the intricate drug release kinetics intrinsic to zein-based ISGs. This validation approach offers a robust basis for the reliability and predictive potential of our zein-based model.

### 3.2. Simulation of Drug Release Pattern by Varying the Rate of Drug Transport from Polymer Matrix to Exterior Phase ($K_{32,k}$) and Drug Release Profile by Varying the Maximum Drug Concentration at the Edge of the Matrix Phase $C_{2,k}\left(r_{2}\right)$

**Figure 5:**
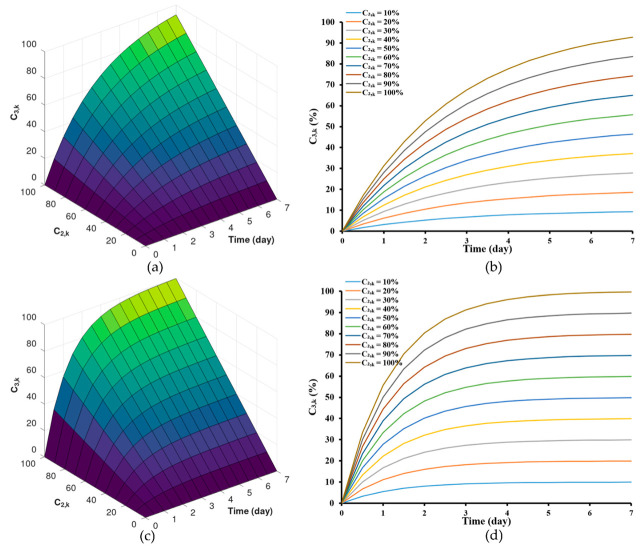
Model predictions of drug release from zein-based ISG formulations for different concentration of drug in the interior at the interface between the interior phase and void space of the matrix, $K_{32,k}$ = 30 (day^−1^) (**a**,**b**) and $K_{32,k}$ = 65 (day^−1^) (**c**,**d**).

As illustrated, this model effectively traced the changes in drug concentrations within the zein-based ISG system over time. This stands in contrast to previously established mathematical models that characterize controlled drug release from matrix systems [61,62,63]. Upon collecting experimental data, the model output was fitted with the experimental data to acquire optimal parameter estimates listed in Table 2. The parameter values were obtained by solving the ordinary differential equation of the model simulations within the specified parameter range. These parameters encompass the rate of drug transport from the polymer matrix to the external phase ($K_{32,k}$). Specifically, $K_{32,k}$ values as indicated at 30 (day^−1^) and 65 (day^−1^). Additionally, the maximum drug concentration at the edge of the matrix phase $C_{2,k}\left(r_{2}\right)\;$ was considered, with $C_{2,k}$ values of 70% and 105%. These parameters, crucial for drug release from the ISG, were determined using the mechanistic model described in Equation (7). The model predictions for drug release from zein-based ISG formulations were assessed for different $C_{2,k}\left(r_{2}\right)$ values, specifically considering $K_{32,k}$ values of 30 (day^−1^) and 65 (day^−1^). These predictions are visualized in Figure 5a,b and Figure 5c,d, respectively.

Although the general initial trends remained consistent with these alterations, it was observed that implants with lower $K_{32,k}$ values (Figure 5a,b) displayed a comparatively slower initial release rate compared to those with higher $K_{32,k}$ values (Figure 4c,d). However, upon reaching a stable steady state in the release of both the solvent and the drug [64], the Lv content in the formulations approached levels close to $C_{2,k}\left(r_{2}\right)$ by the 7th day. Furthermore, the selection of the range of drug transport rates, which spans from 32.55 (day^−1^) to 64.76 (day^−1^), was based on the order *O*(*h*^4^) from Table 2, allowing for a more accurate estimation. For LvZG20 and LvZG25, the rates of drug transport were 32.55 and 50.23 (day^−1^), respectively. Notably, it showed the reduced rates of drug transport at lower concentrations. Additionally, the formation of zein at higher polymer concentrations can be attributed to enhanced molecular chain entanglement, which in turn inhibited the breakup of the polymer jet, leading to sustained release of the loaded drug [65,66]. This observation echoes the earlier finding where the lower molecular weight PLGA implants exhibited a slower initial release rate than the higher molecular weight PLGA implants, with the former demonstrating degradative release at an earlier stage [21]. This approach was mirrored in our selection of ISG formulations, where diverse concentrations of zein were utilized. This deliberate selection aligns with earlier research highlighting the efficacy of varying the concentration of zein in formulations to control the release of low molecular weight drugs [18].

**Figure 6:**
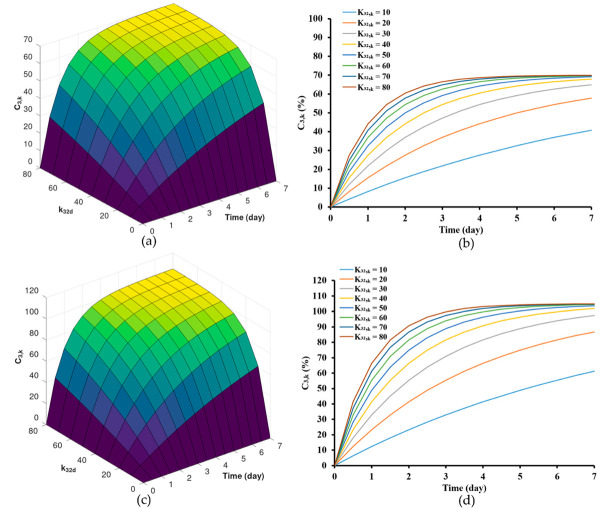
Model predictions of drug release from zein-based ISG formulations for different drug permeability coefficient, $C_{2,k}\left(r_{2}\right)$ = 70 (**a**,**b**) and $C_{2,k}\left(r_{2}\right)$ = 100 (**c**,**d**).

The findings from Table 2 indicate that both LvZD20 and LvZG20 formulations displayed $C_{2,k}\left(r_{2}\right)$ values around 100–105%, while LvZD25 and LvZG25 formulations exhibited $C_{2,k}\left(r_{2}\right)$ values of approximately 70–90%. The model’s estimations of drug release from zein-based ISG formulations were examined for different $K_{32,k}$ values, specifically with regard to $C_{2,k}\left(r_{2}\right)$ values of 70% and 105%. These estimations are visualized in Figure 6a,b and Figure 6c,d, respectively.

The outcomes emphasize the significance of altering the $K_{32,k}$ value. Lower $K_{32,k}$ values were correlated with a comparatively slower initial release rate compared to formulations featuring higher $K_{32,k}$ values. However, during the steady-state phase, the cumulative Lv content gradually approached and eventually reached levels near $C_{2,k}\left(r_{2}\right)$ %. It is noteworthy that formulations with $K_{32,k}$ values ranging from 10 to 40 (day^−1^) did not achieve the $C_{2,k}\left(r_{2}\right)$% within the span of 7 days. Nonetheless, it is evident that these formulations exhibit a tendency to approach the specified threshold, although they require an extended duration to achieve complete release. The concentration of zein significantly impacted the surface and internal topographies, particularly the interconnected porous structure of the ISG [65]. Furthermore, in our previous work, as the amount of zein increased, the pore sizes within the structure notably diminished. These pores served as primary conduits for the release of Lv molecules from the inner zein gel or matrix into the surrounding release medium [9]. This outcome was particularly evident (here or in previous work [9]), indicating that higher zein concentrations were more effective in delaying the drug release.

### 3.3. The Behavior of Lv Diffusion, Solvent Diffusion and Gel Formation

In the typical process, solvent exchange occurred upon the ISGs contact with an aqueous solvent [44]. This initial mechanism within the ISG led to matrix formation and subsequently controlled the drug release. To visualize the mechanisms of the solvent (DMSO and GF), drug (Lv), and matrix-forming component (zein) interactions, an agarose gel system was employed; multiple techniques can be utilized for visualizing these interactions within the gel, including UV-vis imaging, staining, or fluorescent labeling [67,68,69]. UV-vis imaging stands out due to its advantages. Staining techniques, while valuable in certain cases, come with limitations compared to UV-vis imaging. UV-vis imaging offers real-time visualization and quantitative analysis of drug release, ensuring a more accurate and comprehensive tracking of drug behavior within the matrix. Therefore, considering the specific research goals, UV-vis imaging was selected as the more robust method for tracking drug release in this study [39]. The wavelength of 214 nm was selected to monitor the solvents, while a wavelength of 280 nm was chosen to monitor the drug diffusion. Additionally, a wavelength of 525 nm was employed to observe the formation of the solid-like matrix derived from zein, in line with previous studies [40,68,70]. The selection of these wavelengths was motivated by the chromophores of the various components. To this end, GF does not possess a viable chromophore, the apparent change in absorbance was probably reflecting a change in the refractive index. A recognized challenge of UV-vis spectrophotometry, and therefore of UV-vis imaging, is the lack of selectivity as considered below.

**Figure 7:**
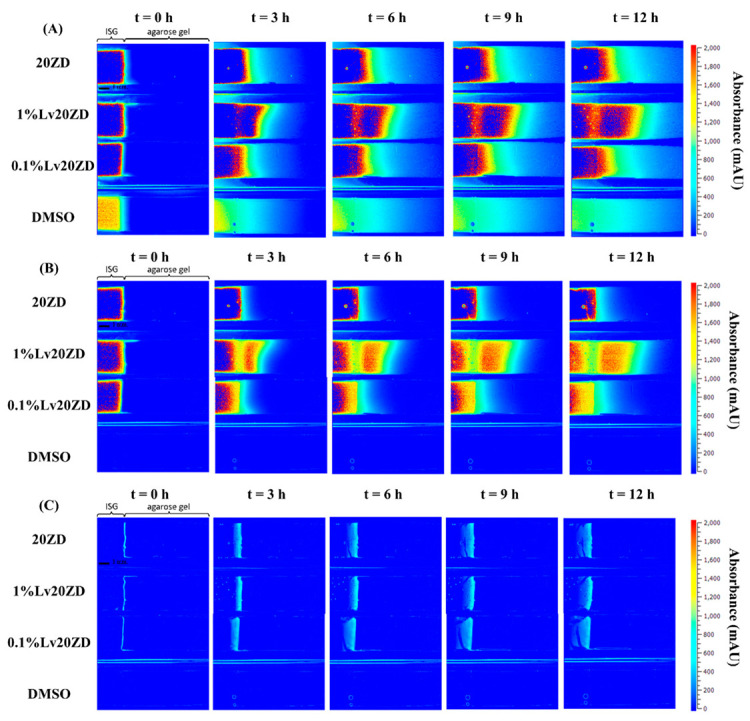
Representative absorbance maps of 20ZD, 1%Lv20ZD, 0.1%Lv20ZD and DMSO observed by UV imaging at 214 nm (**A**), 280 nm (**B**), and visible imaging at 525 nm (**C**) in 0.6% (*w*/*v*) agarose gel at selected time points.

**Figure 8:**
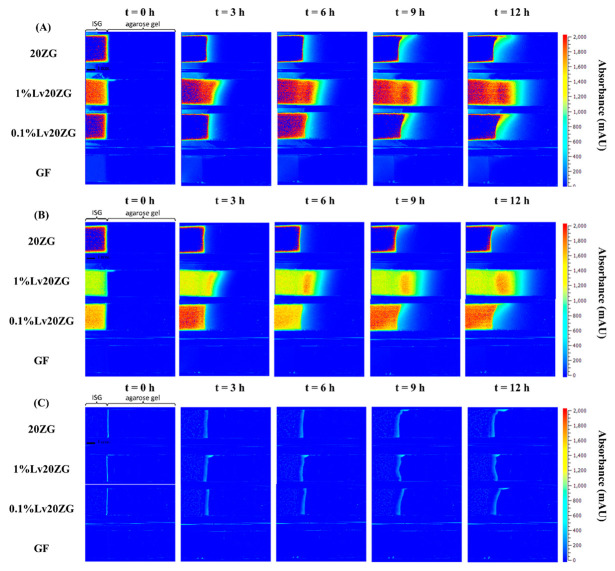
Representative absorbance maps of 20ZG, 1%Lv20ZG, 0.1%Lv20ZG and glycerol formal observed by UV-vis imaging at 214 nm (**A**), 280 nm (**B**), and visible imaging at 525 nm (**C**) in 0.6% (*w*/*v*) agarose gel at selected time points.

Once in contact with aqueous phase from agarose, the ISG promotes diffusion of its solvent (and drug) into the agarose gel; the transport gradually slows down over time as the concentration gradient becomes less steep. The absorbance profile of the solvent on the agarose gel was visualized through color mapping and is displayed in Figure 7A and Figure 8A. The color-coded map depicts solvent absorbances across the agarose gel. Red indicates high concentration, while blue marks near-zero absorbance areas. At the outset, solvent diffusion exhibited its highest rate, gradually diminishing over time. The result might appear similar for both DMSO and ZG20 because, after more than 3 h, formulations containing zein and DMSO exhibited a behavior where the DMSO solvent could permeate the matrix, similar to what was observed with DMSO alone. Therefore, while we did not quantify the concentration, the behavior of DMSO and ZG20 in terms of solvent permeation exhibited similarities beyond a certain timeframe. Indeed, during the initial phase, the matrix formation should occur abruptly, leading to a partial obstruction of solvent diffusion. This obstruction subsequently results in a slowdown of the solvent exchange process [59,65,71]. GF displayed minimal, if any, noticeable absorbance shifts. (Figure 8A), signifying the inadequacy of the 214 nm wavelength for detecting GF. Additionally, it was observed that zein did absorb light at 214 nm [72], as seen in Figure 8A. Over time, the matrix derived from 20ZG exhibited a gradual increase in thickness and firmness. Similarly, in formulations incorporating Lv, the absorbance of Lv at 214 nm was notable [73].

The wavelength of 280 nm was chosen for Lv in this study due to its close alignment with the peak absorbance on the Lv spectrum [74]. Figure 7B and Figure 8B depict the diffusion behavior of Lv through the agarose gel. In Figure 7B at t = 0 h, the coloration that appears as blue corresponds to low absorbance values. However, it is important to consider the potential impact of DMSO interference, which arises from the absorption of DMSO within the spectral range of 289–303 nm [75]. At t = 3 h in Figure 8B, detection of GF was not possible at 280 nm. As depicted in Figure 7B, the vibrant shades of yellow and red (represented using false coloring) in the images correspond to both dissolved drug and drug that has precipitated as previously explained in comprehensive detail [76]. Subsequent to ISG’s contact with the agarose gel, Lv diffusion occurred concurrently with an increasing distance within the gel over time. This behavior is attributed to the solubility of the drug in the zein, solvent and its concentration, which could result in either drug dissolution or dispersion within the in situ forming implant [77]. Depending on these factors, different behaviors have been observed and described. For instance, the impact of low drug loading on the release of in situ implants has been explored in various studies. Wang et al. [78] investigated the effect of ketoprofen loading from 4 to 10% m/m (PLGA 70:30, 35% m/m in NMP) and concluded that it had no significant impact on in vitro drug release. In the case of zein ISG using both DMSO and GF as solvents, it is noteworthy that formulations loaded with 1% Lv, surpassing the 0.1% Lv concentration, exhibited a gradual decline in concentration with increasing distance from the interface. This behavior is attributed to the influence of ingredient concentration on the absorbance profile [79], which might be influenced by zein’s impact on Lv absorbance. This suggests a possible artifact from UV imaging, demanding further investigation for a complete understanding.

In visible imaging at 525 nm, as observed in Figure 7C and Figure 8C, the transformation of zein in DMSO into a matrix appeared to occur more rapidly as compared to GF containing formulations. Additionally, the type of solvent (GF and DMSO) influenced the initiation of gel formation [64]. DMSO, due to its greater polarity and water miscibility in contrast to GF, exerted a stronger effect on the rate and morphology of zein matrix formation, subsequently impacting solvent exchange and controlled drug release rates. The absence of pores within the matrix played a crucial role in determining the drug retention capacity [80]. A well-structured and robust matrix often led to the development of a thick matrix. When a drug was loaded into such a rigid matrix, it became entrapped, resulting in a gradual release over time. Conversely, a less dense matrix could weaken matrix formation and fail to effectively control drug release [9,81]. This outcome reaffirms the previous findings, demonstrating that the utilization of GF as a solvent substantially led to a slower release of Lv compared to DMSO as the solvent [21].

## 4. Conclusions

This study primarily focused on mathematical modeling of drug release from zein-based ISGs. Experimental drug release data were compared with simulation results obtained through numerical differentiation methods. LvZD20 and LvZD25 closely matched experimental data, while LvZG20 and LvZG25 better aligned with experimental data using the forward difference formula of order *O(h*) compared to *O(h^4^)*. Parameters obtained from numerical differentiation were employed to estimate the drug release equation (Equation (7)), offering insights into Lv transport. This study’s innovation lies in the flexible numerical differentiation methods that can handle complex geometries or functions. Findings revealed that higher zein concentrations led to extended drug release, facilitated by denser matrices in high-loading polymers. Moreover, $K_{32,k}$ values suggested LvZD formulations displayed a higher rate of drug transport from the polymer matrix to the exterior phase compared to LvZG. UV-vis imaging performed at 214 nm, 280 nm, and 525 nm visualized solvent, drug, and zein matrix formation processes. The choice of DMSO or GF solvent affected matrix formation rates, influencing solvent exchange and drug release, with GF resulting in slower release than DMSO. These outcomes may contribute to a deeper understanding of controlled drug release from matrix systems and offer methodological advancements for future drug delivery research.

## Figures and Tables

**Table 1:** Composition of Lv-loaded zein-based ISG formulations.

Formula	Levofloxacin HCl (Lv)	Zein	DMSO	GF
	(% *w*/*w*)	(% *w*/*w*)	(% *w*/*w*)	(% *w*/*w*)
Lv20ZD	1	20	79	-
Lv25ZD	1	25	74	-
Lv20ZG	1	20	-	79
Lv25ZG	1	25	-	74
20ZD	-	20	80	-
20ZG	-	20	-	80

**Table 2:** Estimated drug release parameters by numerical differentiation: forward difference formula approximates of order *O*(*h*) and central difference formula of order *O*(*h*^4^).

Formula	Numerical Methods	$K_{32,k}$ (Rate of Drug Transport from the Polymer Matrix to Exterior Phase)	$C_{2,k}\left(r_{2}\right)$ (Drug Concentration at the Edge of the Matrix Phase)
Lv20ZD	*O*(*h*)	56.65	103.50
	*O*(*h*^4^)	52.91	105.50
Lv25ZD	*O*(*h*)	51.57	88.97
	*O*(*h*^4^)	64.76	88.60
Lv20ZG	*O*(*h*)	47.58	98.35
	*O*(*h*^4^)	32.55	104.50
Lv25ZG	*O*(*h*)	34.47	74.01
	*O*(*h*^4^)	50.23	71.58

**Table 3:** Estimated drug release equation by numerical differentiation: forward difference formula of order *O*(*h*) and central difference formula of order *O*(*h*^4^), where $C_{3,k}\left(t\right)$ represents the total quantity of drug released at a specified time (t) (day).

Formula	Forward Difference Formula *O*(*h*)	Rel. Err. *O*(*h*)	r^2^ (O(*h*))	Central Difference Formula *O*(*h*^4^)	Rel. Err. *O*(*h*^4^)	r^2^ (O(*h^4^*))
Lv20ZD	$C_{3,k}\left(t\right)=103.5 [1-e^{0.7081\left(-t\right)}]$	1.34	0.9925	$C_{3,k}\left(t\right)=105.5 [1-e^{0.6614\left(-t\right)}]$	1.36	0.9883
Lv25ZD	$C_{3,k}\left(t\right)=88.97 [1-e^{0.6446\left(-t\right)}]$	1.38	0.9919	$C_{3,k}\left(t\right)=88.6 [1-e^{0.8095\left(-t\right)}]$	1.29	0.9914
Lv20ZG	$C_{3,k}\left(t\right)=98.35 [1-e^{0.5948\left(-t\right)}]$	1.40	0.9894	$C_{3,k}\left(t\right)=104.5 [1-e^{0.4069\left(-t\right)}]$	1.55	0.9770
Lv25ZG	$C_{3,k}\left(t\right)=74.01 [1-e^{0.4309\left(-t\right)}]$	1.52	0.9956	$C_{3,k}\left(t\right)=71.58 [1-e^{0.6279\left(-t\right)}]$	1.37	0.9946

## Data Availability

The data presented in this study are available on request from the corresponding author.

## Supplementary Materials

The following supporting information can be downloaded at: https://www.mdpi.com/article/10.3390/pharmaceutics15102401/s1, Supplement Data S1: discretized using a finite differences method; Supplement Data S2: Source script.
