# Supplementary material for: Numerical Mechanistic Modelling of Drug Release from Solvent-Removal Zein-Based In Situ Gel
Source: Pharmaceutics. 2023 Sep 28;15(10):2401. doi: 10.3390/pharmaceutics15102401 (PMC10609933; doi:10.3390/pharmaceutics15102401)
Supplement: Supplementary file 1 [file pharmaceutics-15-02401-s001.zip › pharmaceutics-2587788-supplementary.pdf]

Supplement Data S1: discretized using a finite differences method

Forward difference formula of order O(h)

For $i = 1,2,3,\ldots,n$ let $y(t_i) = y_i$ and $h = t_{i+1} - t_i$. By Equation (8), the forward difference formula yields

$$\frac{y_{i+1} - y_i}{h} + ay_i = b. \tag{S1}$$

Upon rearranging Equation (S1), the following equation is obtained:

$$(1 - ah)y_i + hb = y_{i+1} \quad \text{for} \quad i = 1,2,3,\ldots,n. \tag{S2}$$

The matrix-vector representation for the linear system (S2) is

$$\begin{bmatrix} y_1 & 1 \\ y_2 & 1 \\ y_3 & 1 \\ \vdots & \vdots \\ y_{n-1} & 1 \end{bmatrix} \begin{bmatrix} 1 - ah \\ hb \end{bmatrix} = \begin{bmatrix} y_2 \\ y_3 \\ y_4 \\ \vdots \\ y_n \end{bmatrix}. \tag{S3}$$

Defined

$$A = \begin{bmatrix} y_1 & 1 \\ y_2 & 1 \\ y_3 & 1 \\ \vdots & \vdots \\ y_{n-1} & 1 \end{bmatrix}, \quad d = \begin{bmatrix} y_2 \\ y_3 \\ y_4 \\ \vdots \\ y_n \end{bmatrix} \; and \; x = \begin{bmatrix} 1 - ah \\ hb \end{bmatrix}.$$

Now, the matrix-vector form is rewritten as follows:

$$Ax = d. \tag{S4}$$

The non-square matrix A with dimensions (n-1, 2) does not have an inverse, preventing direct inversion. Nevertheless, the solution x to Equation (S4) can still be obtained by utilizing the normal equation:

$$A^T Ax = A^T d \tag{S5}$$

where A^T represents the transpose of matrix A.

The solution $x = [x_1 \quad x_2]^T$ can be obtained as $x = (A^T A)^{-1} A^T d$.

Central difference formula of order O(h^4)

Following a similar approach as discussed above, when the central difference formula is employed to approximate the derivative in Equation (S8), a linear system is obtained:

$$\frac{-y_{i+2}+8y_{i+1}-8y_{i-1}+y_{i-2}}{12h}+ay_i=b. \tag{S6}$$

Rearranging Equation (S6), we obtain

$$12hay_i-12hb=y_{i+2}-8y_{i+1}+8y_{i-1}-y_{i-2} \tag{S7}$$

for $i=3,4,5,\ldots,n-2$.

Defined $\Delta_i=y_{i+2}-8y_{i+1}+8y_{i-1}-y_{i-2}$, the matrix-vector representation of Equation (S7) is:

$$\begin{bmatrix} y_3 & -1 \\ y_4 & -1 \\ y_5 & -1 \\ \vdots & \vdots \\ y_{n-2} & -1 \end{bmatrix}\begin{bmatrix} 12ah \\ 12hb \end{bmatrix}=\begin{bmatrix} \Delta_3 \\ \Delta_4 \\ \Delta_5 \\ \vdots \\ \Delta_{n-2} \end{bmatrix}. \tag{S8}$$

Defined

$$A=\begin{bmatrix} y_3 & -1 \\ y_4 & -1 \\ y_5 & -1 \\ \vdots & \vdots \\ y_{n-2} & -1 \end{bmatrix},\quad d=\begin{bmatrix} \Delta_3 \\ \Delta_4 \\ \Delta_5 \\ \vdots \\ \Delta_{n-2} \end{bmatrix}\quad and\quad x=\begin{bmatrix} 12ah \\ 12hb \end{bmatrix}.$$

A solution $[x_1 \quad x_2]^T$ of Equation (S8) is $x=(A^TA)^{-1}A^Td$.

For both the forward difference formula of order O(h) and the central difference formula of order O(h^4), it should be noted that

$$a=\frac{K_{32,k}}{V_3},b=\frac{K_{32,k}}{V_3}C_{2,k}(r_2)\ \ and\ \ \frac{b}{a}=C_{2,k}(r_2)$$

Supplement Data S2: Source script

The scripts for the forward difference formula to approximate the derivative

```
load info.dat
t = info(:,1);
n = length(t);
y = info(:,2);
plot(t,y,'*r','linewidth',2)
h = t(2)-t(1);
E = ones(n-1,1);
A = [y(1:end-1) E];
b = y(2:end);
x = inv(A'*A)*A'*b;
sol_a = (1-x(1))/h
sol_b = x(2)/h
sol_c = sol_b/sol_a
t0 = 0;
time = t0:0.5:7;
y_app = sol_c*(1-exp(sol_a*(t0-time)));
err = norm(y-y_app)/norm(y)
hold on
plot(time,y_app,'--k','linewidth',2)
set(gca,'fontsize',30)
xlabel('{\bf Time (day)}')
legend('Experimental data','Simulation','location','SouthEast')
ylabel('{\bf C_{3,k}(t)}')
```

The scripts for central difference formula to approximate the derivative

```
load info.dat
t = info(:,1);
n = length(t);
y = info(:,2);
plot(t,y,'*r','linewidth',2)
h = t(2)-t(1);
E = - ones(n-4,1);
A = [y(3:end-2) E];
for i = 1:n-4
b(i,1) = -y(i)+8*y(i+1)-8*y(i+3)+y(i+4);
end
x = inv(A'*A)*A'*b;
sol_a = x(1)/(12*h)
sol_b = x(2)/(12*h)
sol_c = sol_b/sol_a
t0 = 0;
time = t0:0.5:7;
```

```
y_app = sol_c*(1-exp(sol_a*(t0-time)));
err = norm(y-y_app)/norm(y)
hold on
plot(time,y_app,':g','linewidth',2)
set(gca,'fontsize',30)
xlabel('{\bf Time (day)}')
legend('Experimental data','Simulation','location','SouthEast')
ylabel('{\bf C_{3,k}(t)}')
```

Script for generating drug release profiles with various $K_{32,k}$ values.

```
V = 80;
t = 0:0.5:7;
k = 0:1:80;
c2= 0:10:100;
[T,C2] = meshgrid(t,c2);
Z = C2.*(1-exp((k(61)/V).*(-T)))
surf(T,C2,Z)
set(gca,'fontsize',30)
xlabel('{\bf Time (day)}  ','fontsize',30)
ylabel('{\bf C_{2,k}}','fontsize',30)
zlabel('{\bf C_{3,k}}','fontsize',30)
save data.dat Z -ascii
load data.dat
```

Script for generating drug release profiles with various $C_{2,k}(r_2)$ values.

```
V = 80;
t = 0:0.5:7;
k = 0:10:80;
c2= 0:10:100;
[T,K] = meshgrid(t,k);
Z = c2(11).*(1-exp((K/V).*(-T)));
surf(T,K,Z)
set(gca,'fontsize',30)
xlabel('{\bf Time (day)}','fontsize',30)
ylabel('{\bfk_{32d}}','fontsize',30)
zlabel('{\bfC_{3,k}}','fontsize',30)
```
